# Rare presentation of 6q16.3 microdeletion syndrome with severe upper limb reduction defects and duodenal atresia

**DOI:** 10.1002/ccr3.916

**Published:** 2017-04-26

**Authors:** Megan L. Donahue, Luis O. Rohena

**Affiliations:** ^1^ Department of Pediatrics San Antonio Military Medical Center San Antonio Texas; ^2^ Division of Medical Genetics Department of Pediatrics San Antonio Military Medical Center San Antonio Texas; ^3^ Department of Pediatrics University of Texas Health Science Center at San Antonio San Antonio Texas

**Keywords:** 6q deletion, diaphragmatic eventration, duodenal atresia, limb reduction, multiple congenital anomalies

## Abstract

We present a patient with a 17.31 MB interstitial deletion of 6q16.3‐6q22.31, who demonstrates a unique constellation of 6q‐ features. Among 6q‐ patients, he has limb reduction among the most severe reported, he is the second patient with duodenal atresia, and is the first documented case of diaphragmatic eventration.

## Graphical abstract

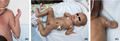

## Introduction

Deletions of the long arm of chromosome 6 are rare, with a little over 100 patients reported in the literature. Hopkin et al. 1 proposed three different phenotypic groups based on location of the deletion using the 60 reported patients with chromosome 6q deletions at that time. Patients with group A (proximal) deletions 6q11‐q16 were found to have higher incidences of hernia, upslanting palpebral fissures, and thin lips. They were less likely to have microcephaly, micrognathia, or heart malformations. Patients with group B (middle) deletions 6q15‐q25 were more likely to have intrauterine growth retardation (IUGR), abnormal respiration, hypertelorism, and upper limb defects. Patients with group C (terminal) deletions 6q25‐qter were more likely to have retinal abnormalities, cleft palate, and genital hypoplasia. Common among all groups were varying degrees of intellectual disability, developmental delays, hypotonia, ear anomalies, and poor postnatal growth 1. In patients with middle chromosome 6q deletions, upper limb defects range from minor to severe deformities, with severe deformities being only rarely reported 1, 2, 3, 4, 5. Here, we present an infant with chromosome 6q16.3q22.31 deletion with severe features including bilateral upper limb reduction defects, duodenal atresia, diaphragmatic eventration, mesocardia, and abnormal facies.

## Clinical Report

The proband is a male infant born via urgent cesarean section at 32 3 of 7 weeks gestation for decreased biophysical profile and nonreassuring fetal heart tones. He was the product of a third pregnancy of a 32‐year‐old mother and 33‐year‐old father. Consanguinity was denied, and the first two pregnancies resulted in healthy daughters.

This pregnancy was complicated by complete placenta previa and prolonged premature preterm rupture of membranes lasting 5 weeks and 1 day. Prenatal ultrasounds beginning at 22 3 of 6 weeks were concerning for duodenal obstruction/duodenal atresia, which was seen on fetal MRI conducted at 28 2 of 7 weeks gestation. Infant was delivered in the breech position and required respiratory support and positive pressure ventilation due to weak respiratory effort. Multiple congenital anomalies were noted at initial examination, and genetics consult was completed on the third day of life.

**Figure 1:**
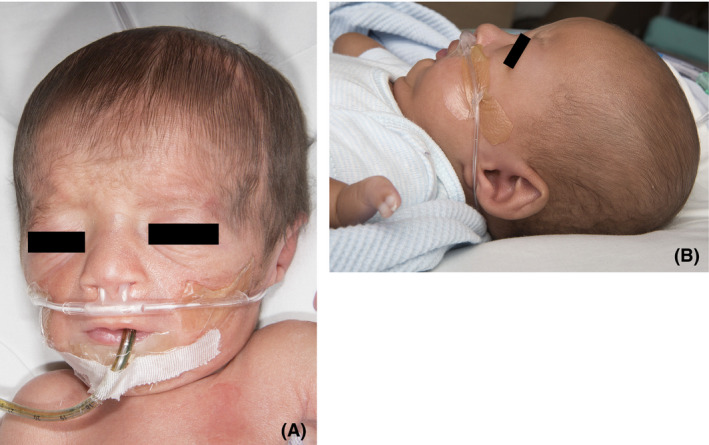
Proband at 5 days (A) and 3 months (B). Note tapered ears, downslanting palpebral fissures, anteverted nares, and micrognathia. The latter image demonstrates bifid nail on left hand. Infant had a persistent supplemental oxygen requirement, ultimately requiring tracheostomy.

Birth weight was 1150 gm (<5th centile), birth length was 43 cm (50th centile), and birth OFC was 27.5 cm (6th centile). Patient was brachycephalic with very large flat anterior fontanel, splaying of the frontal suture, and flattened occiput. He had a low‐lying anterior hairline with hirsute forehead. Palpebral fissures were downslanting. He had no appreciable ocular hypertelorism. Ears were normally positioned but tapered. He had a normal nasal bridge and root with anteverted nares. He had a normal‐length philtrum and vermillion border. There was mild micrognathia without cleft palate (Fig. 1). His neck was short. Cardiac examination was without murmurs. Abdomen was soft and nondistended but was being actively decompressed with replogle. Genital examination showed hypoplastic phallus with bilateral cryptorchidism. Buttocks tissue was atrophic. There was no obvious scoliosis or sacral dimple appreciated. Patient was noted to have mild generalized hypotonia.

**Figure 2:**
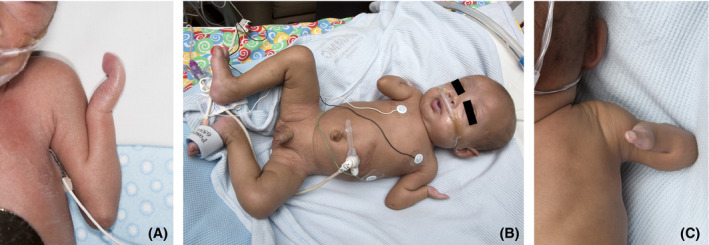
Limb reduction defects. (A) Left arm and hand at 5 days, note webbing of elbow and syndactyly of the two digits. (B) All extremities at 3 months, note posterior rotation of right arm at shoulder, continued webbing of elbows and forearm, and hand reduction with relatively normal lower extremities. (C) View of right arm and hand from behind, showing posterior rotation and single‐digit hand.

Bilateral upper extremities were notable for external rotation of the shoulders with fixed flexion deformity and elbow webbing. He had arm reduction bilaterally with mesomelia and single‐bone forearms. His right hand had a single digit with intact nail. His left hand showed two syndactyly digits with bifid nail. Lower extremities were grossly normal (Fig. 2).

**Figure 3:**
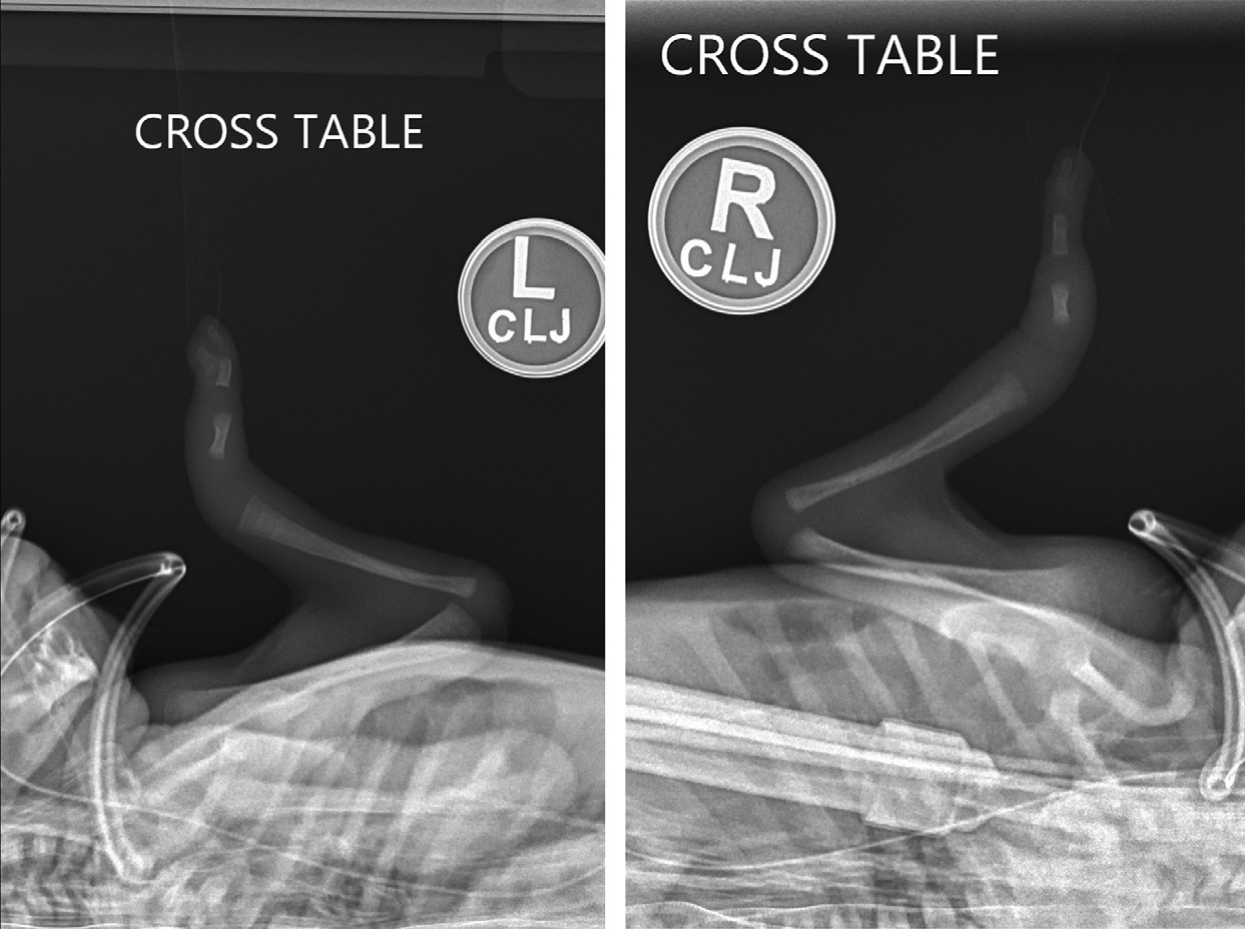
Plain films of left and right forearms, demonstrating absent ulnae, single metacarpals, single proximal phalanges, and parallel distal (left) and bifid distal (right) phalanges.

Imaging of the upper extremities confirmed clinical suspicion of single‐bone forearms with bilateral radii and absent ulnae. The right hand was found to have a single metacarpal, a single proximal phalanx, and a bifid distal phalanx. The left hand was found to have a single metacarpal, single proximal phalanx, and two parallel distal phalanges (Fig. 3).

**Figure 4:**
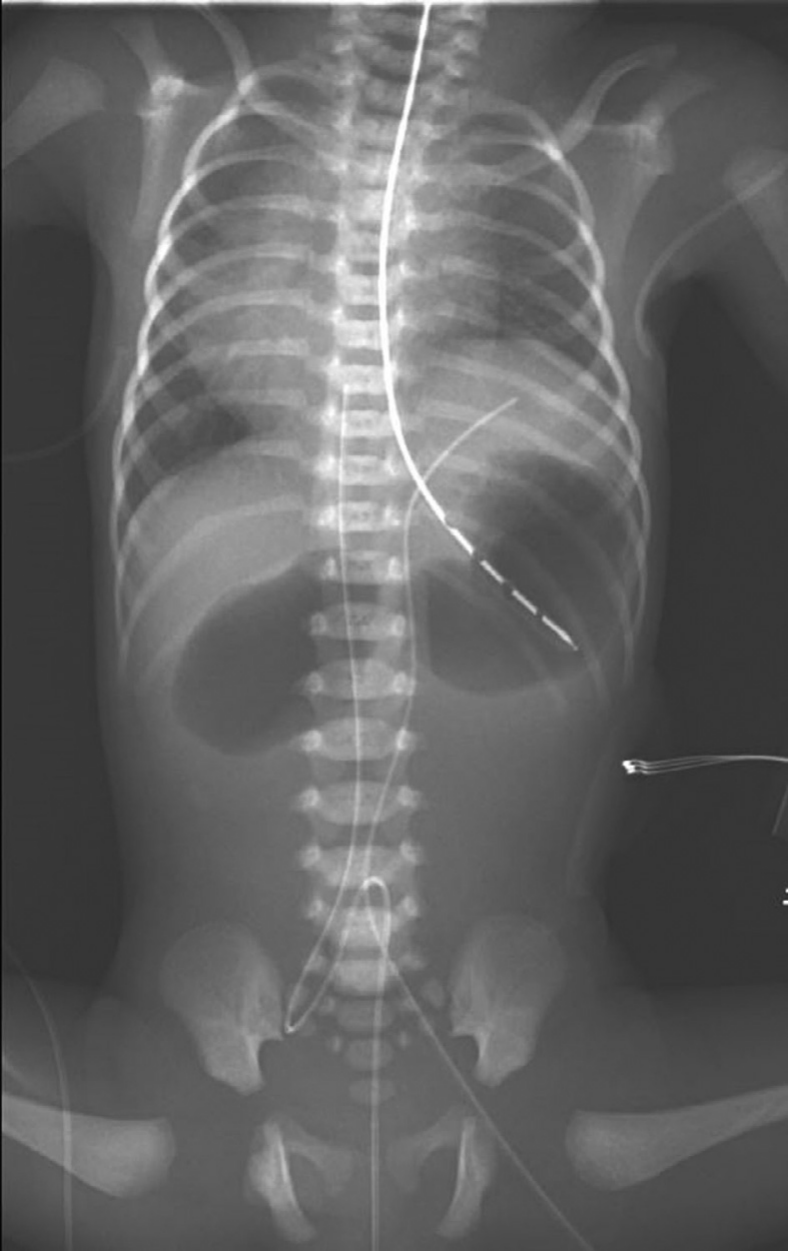
CXR/KUB showing double‐bubble sign of duodenal atresia, elevated left hemidiaphragm. Transposed umbilical venous lines secondary to mesocardia.

Abdominal plain films showed double‐bubble sign concerning for duodenal atresia, which was confirmed on ultrasound day of life 1 (Fig. 4). The patient underwent uncomplicated surgical repair of the atresia on day of life 5. Patient continued to have poor feeding and severe reflux with esophageal and gastric dysmotility. He underwent a fundoplication and percutaneous gastrostomy tube placement at 2 months of age.

**Figure 5:**
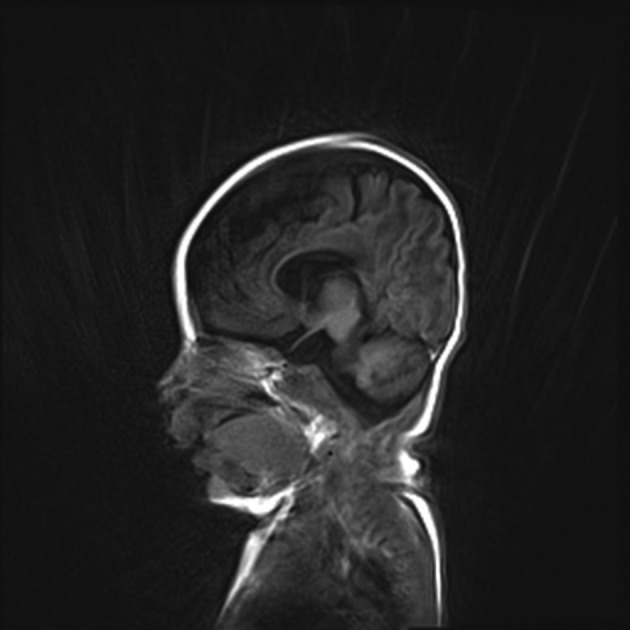
Brain MRI showing ventriculomegaly and thin corpus callosum.

Initial head ultrasound on day of life 1 was concerning for bilateral cysts in the caudothalamic grooves consistent with in utero grade 1 interventricular hemorrhages and left greater than right lateral and third ventricle enlargement. MRI of brain conducted at 1.5 months of life showed continued mild dilation of lateral and third ventricles, mild vermian hypoplasia with megacisterna magna and thin, short corpus callosum (Fig. 5). At 18 months of age, he developed focal epilepsy.

Echocardiogram on day of life 3 revealed mesocardia without transposition, trivial mitral and tricuspid regurgitation, and PFO with left‐to‐right shunt. Spinal ultrasound on day of life 1 showed low position of the conus medullaris at L4 with small lipomas consistent with a tethered cord. In addition to above findings, abdominal ultrasound on day of life 1 also found left renal pelviectasis. Repeat imaging at two months showed no hydronephrosis but bilateral small kidneys both measuring less than 2SD for corrected age.

From birth, patient was noted on serial imaging to have an elevated left hemidiaphragm (Fig. 4). He underwent dynamic studies which demonstrated relative akinesia compared to the right hemidiaphragm consistent with left hemidiaphragm eventration. He underwent plication day of life 27, which required patching at two months of life due to failed plication. Patient continued to have respiratory insufficiency progressing to respiratory failure and required tracheostomy placement at 9 months.

Development of milestones was notably delayed. By 14 months chronological age, patient's CATCLAMS quotient was only 19% of chronologic age, with most notable delays in adaptive skills, scoring at 1.3 equivalent age months.

Initial concern on genetics evaluation was for Cornelia de Lange syndrome, Fanconi anemia, or segmental aneuploidy. Patient underwent *NIPBL*,* SMC1A*, chromosomal breakage and SNP DNA microarray studies. *NIPBL* and *SMC1A* testing were both negative. Chromosomal breakage studies were normal but karyotype demonstrated 46,XY,del(6)(q15q21). SNP DNA microarray using Cytoscan HD Affymetrix 2.67 Million SNP + CNV probes further characterized the patient's deletion as a 17.31 MB interstitial deletion of 6q16.3 with breakpoints of (105,465,388–122,773,040) encompassing numerous genes starting at *LIN28B* and ending at *SERINC1*. FISH testing on parents was normal, showing de novo origin of this patient's deletion.

The content of this manuscript is not considered research at our institution and instead falls into the realm of routine clinical care.

## Discussion

We describe a patient with a middle deletion along chromosome 6 found on SNP microarray studies. The predominant features in our patient include severe upper limb reduction defects, duodenal atresia, diaphragm eventration, mesocardia, and brain anomalies.

Many of the findings seen in our patient overlap with Cornelia de Lange Syndrome (CdLS), specifically the upper limb reduction defects, hirsutism, and cryptorchidism, all of which are classic features of CdLS, although he notably did not have other classic features including synophrys and long philtrum. Given that CdLS has specific gene mutations, he underwent sequencing of the *NIPBL* and *SMC1A* genes, which account for up to 52% of mutations in patients with CdLS. He also underwent breakage studies to evaluate for Fanconi anemia, which can also present with phocomelia, and SNP DNA microarray to evaluate for segmental aneuploidy. Despite clinical similarities to patients with CdLS, *NIPBL* and *SMC1A* testing were negative. Patient was found to have a middle chromosome 6q deletion on both chromosome breakage and SNP DNA microarray studies. Although testing revealed 6q deletion, including both diagnoses in the differential could be important for further patients found to have limb defects and IUGR on prenatal testing.

Our patient demonstrated features found in patients with middle deletions of 6q, although his features were more severe, perhaps due to the size of his deletion. Table 1 compares features of patients with middle deletions of chromosome 6q with those of our patient.

**Table 1:** Comparison of features of proband and previously reported patients with 6q deletions

Case		Chr. Deletion	Abnormal Head Shape	Ear position/shape	Palpebral Fissures	Telorism	Nose Anomaly	Mouth Anomalies	Retro/Prognathia	Neck	Major Upper Limb Anomalies	Minor Upper Limb Anomalies
Nakagome (1980)		6q15q21	Brachycephaly, square face	Lowset posterior rotate	Upslanting	Hypertelorism	+	+	Retro‐		Short/stubby limbs	Tapered fingers
Schwartz (1984)		6q16q22	Macrocephalic		Downslanting	Hypertelorism		+	Pro‐		‐	Tapered fingers with bilateral clinodactyly 5th
Young (1985)	Pt 2	6q21q22		Dysplastic, prominent antihelices	Downslanting		+					Long hands/fingers
Park (1988)		6q22.2q23.1	Metopic and left‐sided craniosynostosis	Small	Transverse							Short 5th metacarpals
Glover (1988)		6q15q21	Brachycephaly, square face, prominent forehead	Lowset posterior rotate	Mongolian	Hypertelorism	+	+				
Horigome (1991)		6q15q21	Microcephaly, brachycephaly, facial flattening	Lowset malformed		Hypertelorism	+	+	Retro‐		Bilateral triphalangia of thumbs	‐
Wakahama (1991)		6q13q21										
Valtat (1992)	Pt 1	6q14q16	Microcephaly		Upslanting		+	+				
	Pt 2	6q14q16	Normocephalic	Lowset	Upslanting		+	+		Short		
Viljoen (1993)		t(6;13)(q21;q12)						+				
Villa (1995)		6q16.2q21	Brachycephaly	Low‐set, posteriorly rotated	Downslanting	Bilateral epicanthus	+	+	Retro‐			Short hands, tapered fingers
Pandya (1995)	Pt 1	6q16.2q23.1	Microcephaly, flattened occiput	Small, asymmetric, malformed	Downslantnig		+	+	Retro‐	Short	Hypoplasia of ulnar and radial rays, hypoplastic/ stiff/ functionless 5th digits, narrow well‐formed thumb, index finger nonopposable with boutonniere deformity, absent digits 3 & 4 bilaterally and absent metacarpals 3 & 4 on right	Hypoplastic nails
	Pt 2	6q16.3q22.3	Wide metopic suture, facial asymmetry, progressive microcephaly		Downslanting	Hypertelorism	+	+			Left hand polydactyly with extra central digit and syndactyly 5/6. Right hand with central ray defect and absent digits 3 & 4	
Stein (1996)		6q22.2q23.1	Microcephaly					+				
Hopkin (1997)	Pt 2	6q16.2q22.32	Brachycephaly, high broad forehead	Lowset, simple, right preauricular sinus	Short	Hypertelorism	+	+	Retro‐		Left: flexion contracture at elbow, single bone left forearm, single digit left hand. Right: four digits with partial syndactyly 2/3, no nail digit 2, abnormal nails digits 3 & 4	Tapered fingers
Gilhuis (2000)		6q15q21	Scaphocephaly, broad forehead		Transverse		+					Short fingers
Le Caignec (2005)		6q15q21	Broad, high forehead, coarse facies	Posterior rotate		Hypertelorism	+	+			‐	‐
Grati (2005)	Pt 1	6del(q22)del(q25.1q25.3)dup(q23q25.1)	Frontal hypertrichosis			Hypertelorism		+	Retro‐	Webbed	Arthrogryposis/joint contractures	5th finger clino
	Pt 3	6q14q16	Narrow bifrontal diameter	Normal set			+	+		Nuchal edema	Flexion deformity right wrist, bilateral camptodactyly digits 2 & 5, proximal implantation of thumbs with broad distal phalanx	
Duran‐Gonzalez (2007)		6q15q22.2	Microcephalic, flat/high forehead	Small	Short	Ptosis, microphthalmia	+	+			Left short arm, central ray defects, adactyly 3/4	Right hypoplastic nails, polyonchia 5
Zherebtsov (2007)		6q16.1q22.32	Flat occiput, heart‐shaped face	Low‐set, posteriorly rotated, malformed	Downslanting	Hypertelorism	+	+	Retro‐	Redundant skin, short/webbed	Ectrodactyly of left hand with 4 fingers	
Klein (2007)	Pt 1	6q16.2q21	Microcephaly, brachycephaly, bitemporal narrowing	Lowset, protruding, simple helix on Right	Downslanting	Hypotelorism	+	+				5th finger clino
	Pt 2	6q16.2q21	Brachycephaly, broad forehead, bitemporal narrowing	Lowset, simple with overfolded helices	Downslanting		+	+	Retro‐			
	Pt 3	6q15q21	Macrocephaly	Lowset		Hypertelorism	+		Retro‐			5th finger clino
Vlckova (2011)	Pt 2	6q14q16	Macrocephaly, low forehead	Dysplastic			+	+	Retro‐			
Rosenfeld (2012)	Pt 1/6.5y	6q21q22.32	Microcephalic, bitemporal hollowing	Ear pits	‐		‐	‐				Extra creases on fingers
	Pt 2/33y	6q21q22.31	Resolved microcephaly	Incompletely folded helix on left	‐		+	+	Retro‐			
	Pt 3/12y		Macrocephalic		Upslanting		‐	‐				Prominent fingertip pads, tapered fingers
	Pt 4/18m	6q21q22.1	Microcephalic, brachycephaly, small AF, bitemporal narrowing	Small, posteriorly rotated, cupped, unraveled helices	Epicanthal folds, downslanting	Hypertelorism	+	+	Retro‐			Camptodactyly at the proximal finger IP joints, absence of 4th distal finger crease, narrow and hyperconvex nails
	Pt 5/10m	6q16.3q22.31	Microcephalic, brachycephaly	Unraveled helices	Downslanting		+	+				Middle finger camptodactyly, hyperconvex nails
	Pt 6/12y	6q21q22.1				Hypertelorism	+	‐				Long and slender fingers
	Pt 7/5y		Microcephalic		‐		‐	‐			‐	‐
	Pt 8/23y		Microcephalic, short forehead, oval facies	Flattened helix	Upslanting	Hypotelorism	+	+	Retro‐		‐	‐
	Pt 9/12y	6q16.1q21	Prominent forehead	Hypoplastic anterior helix	Upslanting		+	+			‐	Small hands, 5th finger clinodacyly, brachydactyly
	Pt 10/3y	6q15q16.3	Macrocephalic		‐		‐	+			‐	Tapered fingers
	Pt 11/11y	6q16.2q16.3	Microcephalic	Small	Short, upslanting		+	+			‐	‐
	Pt 12/11y	6q16.2		Cupped, dysplastic	‐		+	‐			‐	‐
Izumi (2013)		6q16.1q21			Downslanting, small		+				Short upper limbs	5th finger clino, small middle phalanx
Vignoli (2013)		6q16.1q21	Long face, low anterior hairline	Small, lowset, posterior rotate	Upslanting		+	+	Pro‐	Short		Small hands
Donahue (2017)	3 days	6q16.3q22.31	Macrocephaly, brachycephaly	Dysplastic	Downslanting	Normal	+	‐	Retro‐	Short	Severe reduction defects as described in text	

Case	Lower Limb Anomalies	Scoliosis	Hypogenitalia	Hypotonia	Respiratory Insufficiency	Cardiac Defects	Gastrointestinal	Brain Anomalies	SNHL	Poor Vision	Seizures	Other
Nakagome (1980)	+		‐	+	+	+						
Schwartz (1984)	‐	+		+				+			+	Developed hypertonicity age 11
Young (1985)	+	#REF!								+		
Park (1988)				+								
Glover (1988)			+	+								Pectus carinatum, umbilical hernia
Horigome (1991)	‐			+	+	+		+				
Wakahama (1991)		+			+	+		+				Multicystic kidneys (Potter IIb)
Valtat (1992)												Short stature
				+								
Viljoen (1993)	+									+		Ectrodactyly of both feet
Villa (1995)				+				+		+		Obesity, PWS‐like features
Pandya (1995)	+		+	+		+	Duodenal atresia		+	+		Anteriorly‐placed anus, hemiplastic thoracic
	+			+		+		+		‐		
Stein (1996)			+	+		+			+		+	Obesity without food‐seeking behavior, gynecomastia, GH deficiency
Hopkin (1997)	+		+		Prolonged mechanical ventilation, anomalous subclavian artery compressing trachea, laryngomalacia	+	Gastrostomy requirement	+	+	‐	+	
Gilhuis (2000)	+			+		+		+		+		Hypothyroid, GH deficiency
Le Caignec (2005)	‐		‐	+		‐		‐				
Grati (2005)	+	+		+	+	+	Immature bowel	+				2V cord, death at 22wk EGA, fetal akinesia
	+											Blind‐ending sacral sinus
Duran‐Gonzalez (2007)	+				Pulmonary HTN	+	Ileocolic volvulus					
Zherebtsov (2007)	+				+	+		+				Hypothyroid
Klein (2007)	+			+				+		+		Umbilical hernia, gynecomastia
				+						+		Hemangioma over posterior fontanelle, retinitis pigmentosa
	+									+		Obesity, diabetes insipidus
Vlckova (2011)			+		Laryngomalacia, congenital diaphragmatic hernia	+	Congenital diaphragmatic hernia	+		+	+	Bl iris colobomata, optic disc hypoplasia, hypermobile joints
Rosenfeld (2012)	+		‐	‐		‐		NA		‐	+	
	+	+	+			‐		+		+	+	Gynecomastia, hyperextensible joints
	+		‐	+		‐		+		‐		Hip laxity
			‐	‐		+		+		+	‐	Small chest wall musculature
	+		‐	+		+	Delayed gastric emptying, GERD	+			+	rAOM, nasolacrimal duct stenosis
	+		+	‐		‐		‐			‐	Hyperextensible joints
	‐		+	‐		‐		+			+	
	+		Bifid uterus	‐		‐		‐			+	Hypothyroid
	+		+	+		‐		+			‐	Bifid uvula, submucosal cleft, hyperreflexive bladder
	‐		‐	‐		‐	GERD	+			‐	Sleep apnea, hypothyroidism
	+		‐	+		‐		+			+	
	‐		‐	‐		‐	Bowel blockage at 4 months	‐			‐	Velopharyngeal insufficiency
Izumi (2013)	+		+	+								Obesity, GH deficiency, hypothyroid
Vignoli (2013)	+	+		+		+		+			+	Hand stereotypies
Donahue (2017)	‐	‐	+	+	Left hemidiaphragm eventration	+	Duodenal atresia	+	+	+	+	

Limb anomalies are well‐documented in patients with deletions of 6q between bands 15 and 25. However, many of these defects are minor, including short hands, short and/or tapered fingers, fifth finger clinodactyly, or hypoplastic nails. Reports of patients with major anomalies of the upper extremities have been rarer. Pandya et al. 4 report two patients with significant reductions including radial or ulnar hypoplasia with loss of digits. Duran‐Gonzalez et al. 2 and Zherebtsov et al. 5 also report patients with split‐hand deformities. Grati et al. 3 report two patients with contraction deformities of the upper limbs, one of which also had camptodactyly and proximal implantation of the thumb. Case 2 reported by Hopkin et al. 1 showed reduction defections most similar to those of our patient with flexion contracture at the elbow, a single arm forearm, and a single‐digit hand of the left upper extremity. The length of the deletions of all of these patients, except that of who had a complex deletion/duplication syndrome, is all similar to that of our patient extending 6–7 bands and ranging from 6q15 to 6q23. The greater amount of deleted genetic material in these patients may have contributed to the more severe limb anomalies, although a few other patients with similar deletions have only minor defects. Limb reduction defects are not seen in patients with proximal or distal chromosome 6q deletions.

Although many patients have been reported as having some form of respiratory difficulties, most of these have involved perinatal respiratory insufficiency requiring assistive ventilation. Few have been reported to have severe peripheral anatomic airway disease. Hopkin et al. 1 report a patient with prolonged mechanical ventilation in the setting of laryngomalacia and anomalous subclavian artery suppressing the aorta. Duran‐Gonzalez et al. 2 report a patient with pulmonary hypertension and Vlckova et al. 6 report a patient with laryngomalacia and congenital diaphragmatic hernia. To our knowledge, our proband is the first reported patient with congenital diaphragmatic eventration (CDE). Eventrations have been rarely reported in deletion syndromes along other chromosomes, including chromosome 3 7, in trisomies 13 and 18, and in Fryns syndrome, although diaphragmatic eventration has uncertain genetic etiology. CDE can present in the neonatal period as respiratory distress including tachypnea, accessory muscle use and cyanosis potentially progressive to respiratory failure. If found in other patients with middle chromosome 6q deletions, CDE may represent an important diagnostic consideration in 6q‐ patients presenting with respiratory distress.

To our knowledge, duodenal atresia has only been reported one other time in a patient with a chromosome 6q deletion. The first case of Pandya et al. 4 described a patient with a deletion 6q16.2q23.1, similar to our patient, who also had duodenal atresia and limb reduction defects, as mentioned above. This patient also underwent surgical repair of her atresia, but aside from continued poor growth, no mention was made of her further gastrointestinal course. Our patient, like many others with similar deletions, has had poor feeding and required placement of a gastrostomy tube. While common in duplication syndromes like Down Syndrome, which accounts for 24% of cases in some series 8, duodenal atresias are very rarely seen in other deletion syndromes with isolated cases reported in deletions of chromosomes 2p, 2q, 11q, 9q, and 17q. Duodenal atresias are also seen occasionally in other syndromes including Fanconi Pancytopenia Syndrome, Fetal Hydantoin Syndrome, and Fryns syndrome. Specific genetic etiology is uncertain. Further reports of large segment 6q deletion syndrome patients may uncover more patients with this anomaly and point to an identifiable susceptibility locus.

Similar to many patients identified in our literature review, our patient presented with cerebellar hypoplasia and ventriculomegaly. Of 40 patients reviewed, 21 had reported brain anomalies, four were reported to have normal brain imaging, and no mention was made in 15. Of the 21 with anomalies, seven (33.3%) had corpus callosum abnormalities, of which five (23.8%) were complete or partial agenesis or hypoplasia. Four patients (10%) were noted to have enlargement of ventricles, most commonly lateral and/or third. Also reported were periventricular leukomalacia (7.5%), cerebellar hypoplasia, or atrophy (7.5%) and generalized brain atrophy (7.5%). Additionally, our patient had later development of epilepsy. Stereotypic epilepsy syndromes have previously been described in patients with 6q deletions, especially terminal deletions 9. Similar structural findings in patients with 6q deletion may help further characterize risks or loci for these epilepsy patients.

## Authorship

MLD: involved in study conception and design, acquisition of data, analysis and interpretation of data, drafting of manuscript, critical revision. LOR: involved in study conception and design, acquisition of data, analysis and interpretation of data, drafting of manuscript, critical revision.

## Conflict of Interest

None declared.
